# Radiosynthesis and *in-vitro* identification of a molecular probe ^131^I-FAPI targeting cancer-associated fibroblasts

**DOI:** 10.3389/fonc.2024.1442601

**Published:** 2024-08-30

**Authors:** Yaxin Tian, Yanghongyan Jiang, Ping Ma, Xiaowei Ma, Liang Du, Fengkui Wang, Xiaodong Yu, Qian Zhao

**Affiliations:** ^1^ Department of Nuclear Medicine, General Hospital of Ningxia Medical University, Yinchuan, Ningxia, China; ^2^ School of Clinical Medicine, Ningxia Medical University, Yinchuan, Ningxia, China; ^3^ Department of Nuclear Medicine, The Second Affiliated Hospital of Guangzhou Medical University, Guangzhou, Guangdong, China

**Keywords:** cancer-associated fibroblasts, FAPI, molecular probe, ^131^I, identification

## Abstract

**Purpose:**

Fibroblast activation protein (FAP) is highly expressed in the mesenchyme of most malignant epithelial tumors, while its expression is low in normal tissues. FAP inhibitors (FAPIs) bind specifically to FAP and are used for tumor-targeted diagnosis and therapy. The aim of this study was to radiosynthesize a novel molecular probe ^131^I-FAPI and evaluate its *in-vitro* targeting and biological characteristics.

**Methods:**

The structurally modified FAPI was labelled with ^131^I through the chloramine-T method. The radiolabeling rate was then detected by thin-layer chromatography (TLC). The stability of ^131^I-FAPI was determined at PBS (room temperature) and serum (37°C). Its hydrophilicity was calculated by measuring its lipid-water partition coefficient. Pancreatic cancer PANC-1 cell line and glioma U87 cell line were cultured *in vitro*. Cell uptake assay was used to show the binding ability of ^131^I-FAPI. The CCK-8 assay was used to calculate the inhibitory effects of ^131^I-FAPI at different time points (4h, 8h, 12h, 24h, 48h) after comparing with the ^131^I and FAPI. The before-and-after-24h scratch areas of the two cells were determined in order to verify the effect of ^131^I-FAPI on the migration ability of the cells.

**Results:**

The radiolabeling rate was (84.9 ± 1.02) %. The radiochemical purity of ^131^I-FAPI remained over 80% in both 25°C PBS and 37°C serum. The value of the lipid-water partition coefficient was -0.869 ± 0.025, indicating the hydrophilic of the probe. The cellular uptake assay showed that U87 cells had a specific binding capacity for ^131^I-FAPI. In cell inhibition assays, the inhibitory effect of ^131^I-FAPI on U87 cells increased with time. The results of cell scratch assay showed that ^131^I-FAPI had the strongest inhibitory effect on the migratory ability of U87 cells compared with ^131^I and FAPI (*P*<0.001).

**Conclusion:**

^131^I-FAPI was synthesized with good *in-vitro* stability and hydrophilic properties. It can be specifically bound by U87 cells. The proliferation and migration of U87 cells can be effectively inhibited. ^131^I-FAPI is promising to become a therapeutic probe.

## 1 Introduction

Recently, the hotspot of tumor target is the tumor microenvironment (TME). Because tumors are not isolated masses in organs, but are caused by various complicated factors in TME (1). TME is a complex internal environment that consists of different cell types, vessels, lymphatic vessels, and some physical elements. Cancer-associated fibroblasts (CAFs) are one of the most important ingredients. As the main stromal cells, CAFs promote the invasion and migration of tumors through several mechanisms. Therefore, targeting to CAFs can suppress the growth and progress of tumors.

Fibroblasts upregulate the expression of certain molecules after activating to CAFs, such as, myostatin C, platelet-derived growth factor receptor α or β, fibroblast activating protein (FAP), and natriuretic peptide B and so on (2). Among them, FAP is highly specific for expression on 90% tumor stroma (e.g, primary and metastatic cancers of the breast, lung, and colorectal cancer), and almost not expressed in benign tumors and normal tissues. So FAP is regarded as a specific marker of CAFs. In other words, the expression level of FAP plays an important role in the diagnosis of malignant tumors, staging and recurrence. Most importantly, molecular probes targeting FAP can be specifically located in tumors to achieve tumor imaging and treatment.

In recent years, radionuclide labelled probes targeting FAP were widely used in tumor diagnosis and treatment. Based on the molecular imaging, the pathophysiological processes in molecular, cellular or subcellular level can be visualized and quantified. At the same time, the information of the structure and function will be obtained. Among radionuclide imaging devices, positron emission tomography/computed tomography (PET/CT) is more sensitive for the visualization of tumors. But it has not yet been fully popularized in the primary hospitals due to the high cost of positronic tracer. In this way, the use of single-photon radionuclides for research on single photon emission computed tomography/computed tomography (SPECT/CT) scan is extremely necessary.

Fibroblast activation protein inhibitor (FAPI) specifically binds with FAP and can be selectively expressed in FAP-positive tissues. It was previously used as a class of anticancer drugs (3). But now as a novel target, it has been developed to be labeled with nuclides for tumor nuclide imaging and therapy in nuclear medicine. ^68^Ga-labeled FAPI PET/CT imaging is the most widely used, and has the advantage of evaluation on primary and metastatic foci of a variety of tumors compared with ^18^F-FDG PET/CT imaging (4, 5). Subsequent studies on ^18^F/^99m^Tc-labelled FAPI have also confirmed the feasibility of this type of molecular probe for imaging (6, 7). Our group synthesized a novel FAP-targeting SPECT/CT imaging molecular probe ^99m^Tc-HYNIC-FAPI. It has a high radiolabeling rate and good *in-vitro* stability with specific uptake ability for tumor cells overexpressing FAP (8).

In order to explore the potential of FAPI-based radionuclide therapy, several teams developed therapeutic molecular probes. ^177^Lu-FAPI (9) has achieved preliminary results in targeted radioligand therapy (TRT) for tumors. Studies on ^90^Y (10), ^64^Cu, ^225^Ac (11), ^153^Sm (12) labeled FAPI were only case reports, but they showed the effectiveness in tumor therapy with the need of more verifications on their therapeutic efficacy and toxicity. However, the above nuclides are difficult to obtain and expensive and requires special equipment.

As the earliest and widely used single-photon nuclide, ^131^I emits β and γ rays with greater availability, longer biological half-lives, and greater operability. For primary hospitals, it plays vital roles on disease diagnosis, treatment, and biomedical researches as a labelled nuclide. The selection of ^131^I will be more conducive to promoting the application of FAPI research in treatment, and has good clinical translation value. However, there were few studies on ^131^I labeling FAPI (13).

The purpose of this study was to explore a new molecular probe, ^131^I-FAPI. The stability and lipophilic properties of the probe *in vitro* were evaluated. The specific binding ability of the probe on tumor cells was verified through cell uptake experiments. The cell proliferation toxicity assays and cell scratch assays were used to confirm the inhibition of the probe on tumor cell proliferation and migration, which laid a foundation for therapeutic assessment based on tumor-bearing models.

## 2 Materials and methods

### 2.1 Preparation of the ligand

**Figure 1:**
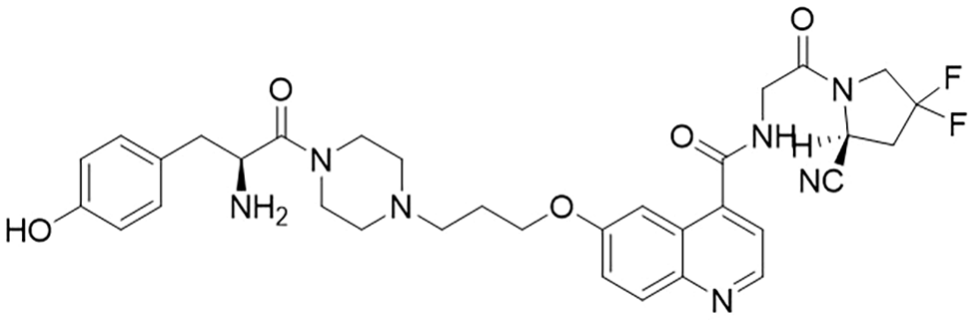
The structure of the new ligand of ^131^I-FAPI.

H-Tyr-FAPI ligand was designed and synthesized from Ganzhou Probe Biopharmaceutical Co., Ltd (Jiangxi, China). **Figure 1** is the structure of the FAPI, which was modified with tyrosine based on FAPI-04. Its purity was >95% determined by high–performance liquid chromatography (HPLC). The peptide was diluted in phosphate-buffered saline (PBS) at the concentration of 2mg/ml before being stored at -20°C.

### 2.2 Radiolabeling of FAPI with ^131^I

In this study, the radiolabeling method was Chloramine T method. ^131^I and chloramine-T trihydrate and Sodium metabisulfite were obtained from Sichuan CNNC Qualcomm Pharmaceutical Co., Ltd. and Shanghai Macklin Biochemical Co., Ltd., respectively. The varying mass of FAPI (50μg, 60μg, 70μg, 80μg and 90μg), the concentration of Chloramine T (1mg/ml, 1.5mg/ml, 2mg/ml, 2.5mg/ml, 3mg/ml, 4mg/ml and 5mg/ml) and the reaction time (2min, 5min, 10min, 15min and 30min) were used to obtain optimal conditions of radiolabeling.

Thin layer chromatography (TLC) was used for the determination of radiolabeling rate.

### 2.3 Stability of ^131^I-FAPI

^131^I-FAPI was mixed with different medium, 25°C PBS and 37°C fetal calf serum that mimics the human environment. At different time points (0h, 4h, 8h, 24h, 48h, 72h), the radiochemical purity and the stability of the probe were determined with paper chromatography. The results were calculated in GraphPad Prism v9.

### 2.4 Partition coefficient

The hydrophilicity or lipophilicity of ^131^I-FAPI was obtained by the following steps. 50µl ^131^I-FAPI, 500µl PBS and 500µl n-octanol were added into a centrifugal tube. The tube was vibrated on a vortex mixer (Thermo Scientific, USA) for 2 min and then centrifugated at 13000 rpm for 5 min. Samples were separately collected from lipid phase and aqueous phase. The radioactivity was counted with automatic gamma counter (PerkinElmer Instruments Inc., USA).

### 2.5 Cell culture

The human glioblastoma cell line (U87MG) and the pancreatic cell line (PANC-1) (Suzhou Haixing Biological Technology Co., Ltd, China) were cultivated in Dulbecco modified Eagle medium (DMEM) supplemented with 10% fetal bovine serum (FBS) and 1% penicillin streptomycin at 37°C under 5% CO_2_.

### 2.6 Cell uptake essays

Three groups were divided, including ^131^I-FAPI group, ^131^I group and blocking group (FAPI was added 30min before adding ^131^I-FAPI). U87 cells were seeded in 24-well plates (5×10^4^ cells/well) for 24h to 80%-90% density (3 wells per group). They were incubated in FBS-free DMEM containing the three different agents for another 24h. The medium and cells were separately collected before measuring their radioactivity counts with γ counter. Cell uptake rate=100% × radioactivity counting of intracellular/(intracellular + extracellular). The uptake of ^131^I-FAPI on PANC-1 cells was verified using the above same process. The experiment was done in triplicate.

### 2.7 Cell proliferation and cytotoxicity essays

Cells were seeded in 5 96-well plates at the concentration of 8×10^3^ cells per well and cultured for 24 hours. Then the medium was replaced with 100µl FBS-free DMEM containing ^131^I-FAPI, ^131^I, FAPI or PBS (3 wells per group). Cells were cultivated for 5 time points, 4h, 8h, 12h, 24h, 48h at 37°C. At each time point, 10µl Cell counting Kit-8 (CCK8) was added into each well. The optical density (OD) at 450 nm was measured after two-hour incubation of medium with CCK8 in the dark place. The survival rate of cells was then calculated in GraphPad Prism v9.

### 2.8 Cell scratching assay

Cells were seeded in 6-well plates. After being cultivated for 24h, cells were washed, scratched and then incubated in FBS-free DMEM containing ^131^I-FAPI, ^131^I, FAPI or PBS for 24h at 37°C. Cell growth was recorded at 0h and 24h after cells were scratched using a 200μl plastic pipette tip.

### 2.9 Statistical analysis

Data analysis was performed using SPSS (IBM, version 26.0). The quantitative data were presented as mean ± standard deviation (SD). The differences between groups were compared using Student’s t-test and one-way ANOVA. *P* < 0.05 were considered statistically significant.

## 3 Results

### 3.1 Synthesis and radiochemistry

**Figure 2:**
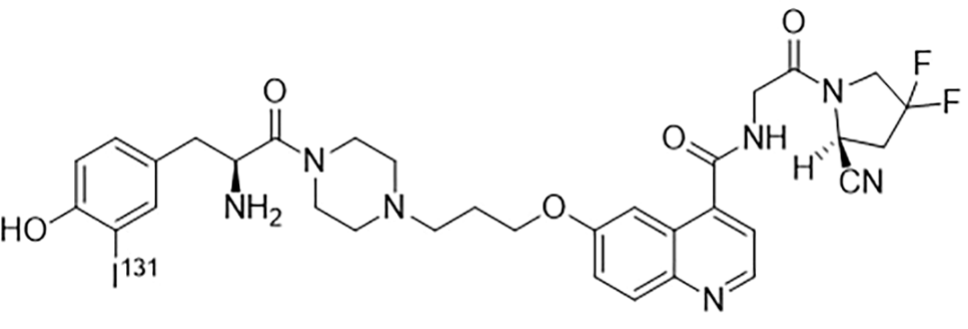
The structure of ^131^I-FAPI.

By single variable control method, optimal radiolabeling condition was determined: 37MBq fresh iodine was dropped into 35µl 70µg FAPI solution. Then 100µl 1.5 mg/ml freshly prepared Chloramine T and 50µl PBS were added. The mixture was vortexed for 15 min before the reaction was terminated by 100µl 1mg/ml Sodium metabisulfite. The structure of ^131^I-FAPI was shown in **Figure 2**.

**Figure 3:**
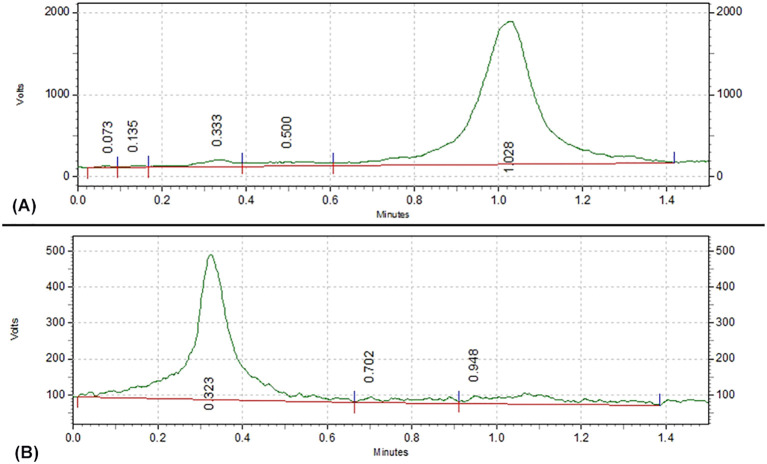
**(A)** TLC of pure ^131^I; **(B)** TLC of ^131^I-FAPI.

When NaCl was used as the mobile phase, the radiolabeling rate of ^131^I-FAPI was (84.9 ± 1.02) %. As shown in **Figure 3**, ^131^I-FAPI and ^131^I were expanded at totally different position.

**Figure 4:**
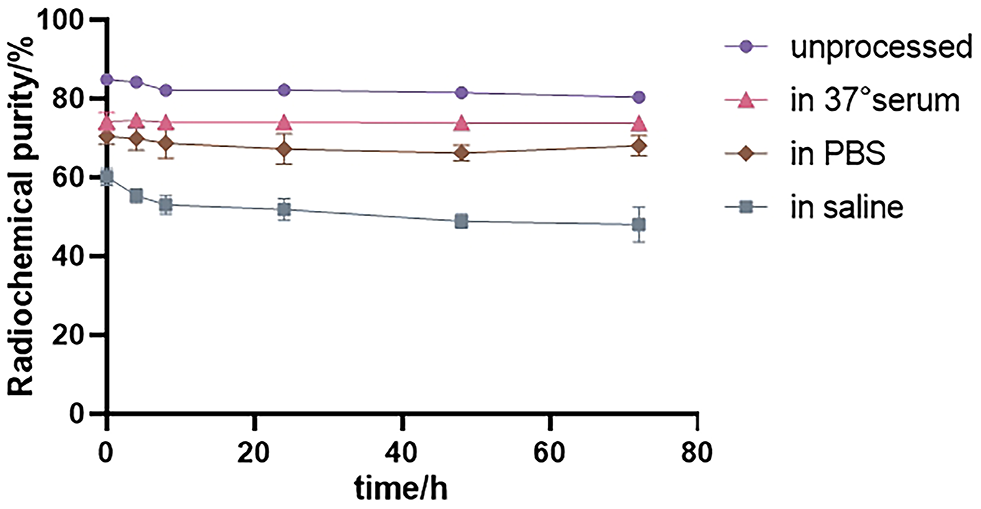
Radiochemical purity and stability of ^131^I-FAPI in different conditions.

As shown in **Figure 4**, the radiochemical purity of ^131^I-FAPI was still more than 80% over time, but it decreased slightly in different environments. It was higher in 37°C serum than in other conditions. In saline, the radiochemical purity was the lowest.

The partition coefficient (log P) value of ^131^I-FAPI was -0.869 ± 0.025, indicating the hydrophily of the probe.

### 3.2 Cellular uptake of ^131^I-FAPI

**Figure 5:**
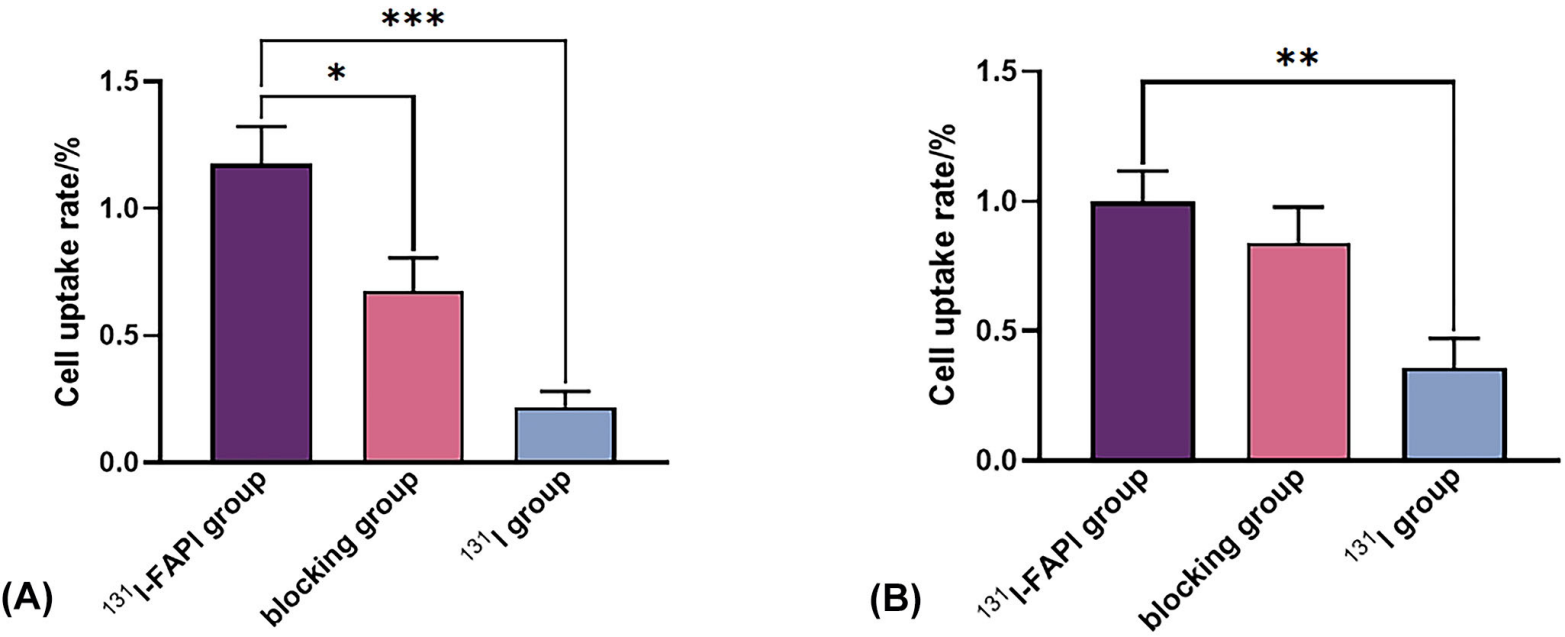
**(A)** cell uptake rate of U87 cells; **(B)** cell uptake rate of PANC-1 cells. *, *P*<0.05; **, *P*<0.01; ***, *P*<0.001.

After 24-hour drug intervention in each group, the uptake rate of U87 cells was the highest in the ^131^I-FAPI group, which was (1.18 ± 0.29) %. The uptake rates in the blocking group and the ^131^I group were (0.68 ± 0.26) % and (0.22 ± 0.12) %, respectively. As shown in **Figure 5**, there was a significant difference between the ^131^I-FAPI group and the ^131^I group (*P*<0.001). For PANC-1 cells, the uptake rates in ^131^I-FAPI group, blocking group and ^131^I group were (0.99 ± 0.12)%、(0.84 ± 0.14)% and (0.36 ± 0.12)%, respectively. A statistical difference was only shown in ^131^I-FAPI group and ^131^I-FAPI group (P<0.01).

### 3.3 Inhibition of ^131^I-FAPI on cell proliferation and migration

**Figure 6:**
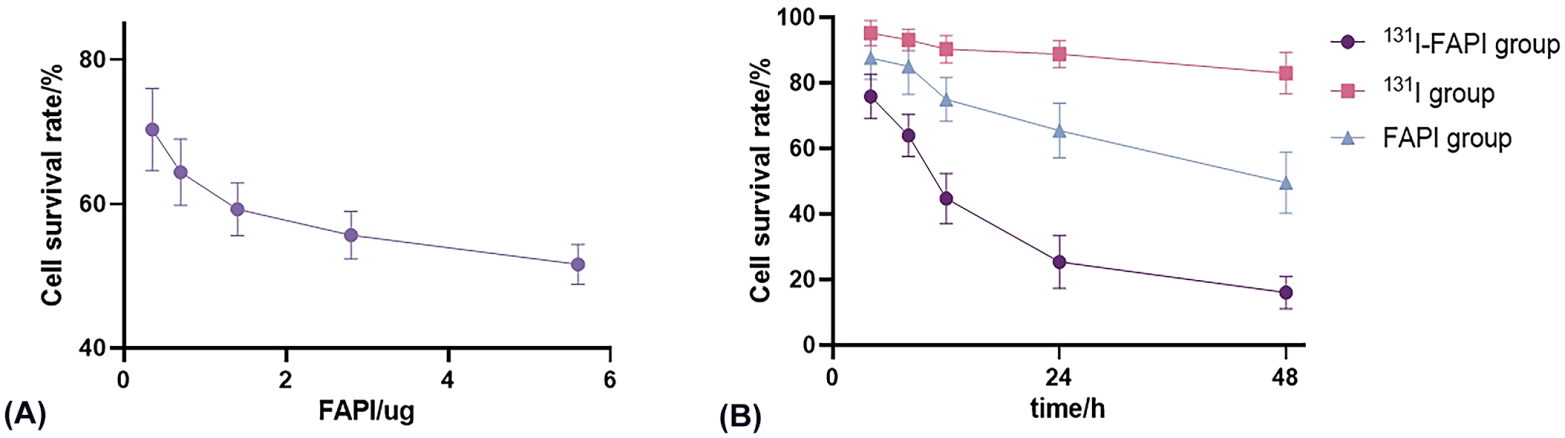
**(A)** Effect of concentration of FAPI on the viability of U87 cells. **(B)** Effect of incubation time with ^131^I-FAPI, ^131^I, FAPI on the viability of U87 cells.

After measuring the OD value at 450 nm, we found that in all groups, the viability of U87 cells decreased over time (**Figure 6**). The inhibition of ^131^I-FAPI on the cell viability was the highest. In addition, after drug intervene of 12h, 24h, 48h, cell survival rate significantly lowered in ^131^I-FAPI group compared with ^131^I group (*P*<0.01). But for PANC-1 cells, the cell survival rate fluctuated.

**Figure 7:**
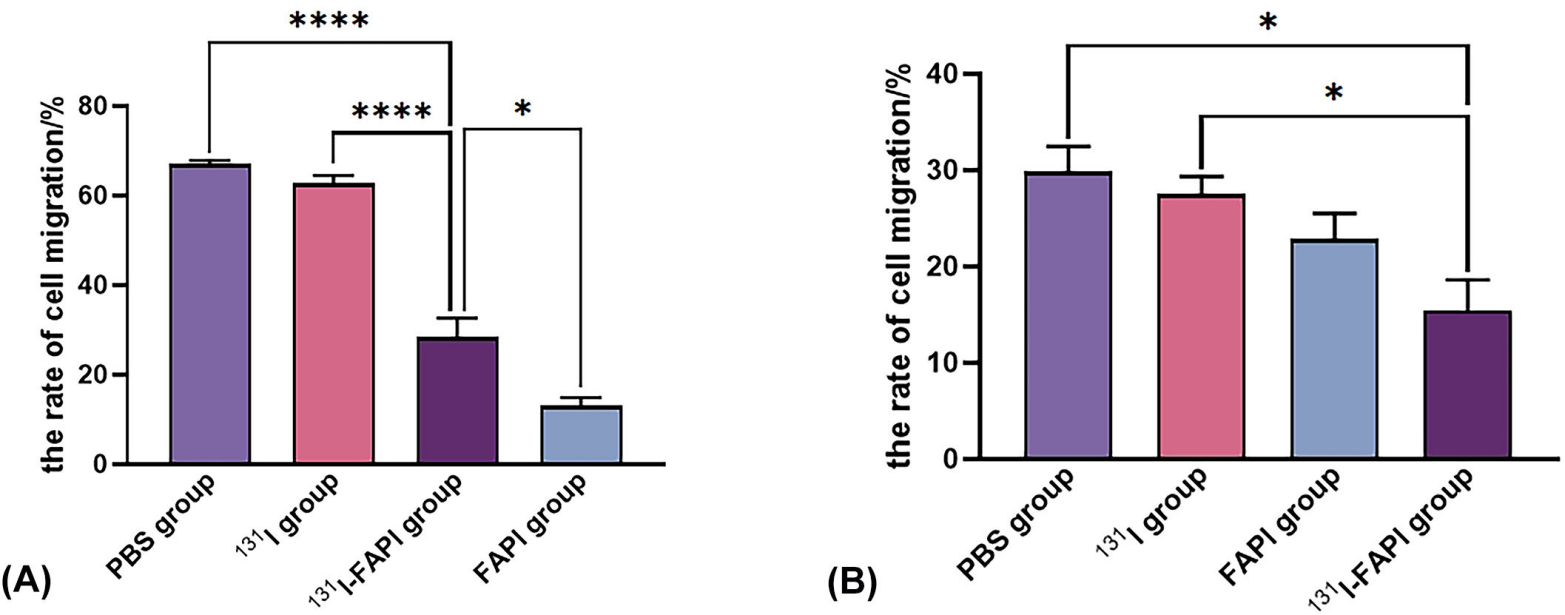
The rate of cell migration on U87 cells **(A)** and PANC-1 cells **(B)** after interventions with ^131^I, ^131^I-FAPI, FAPI and PBS. *, *P*<0.05; ****, *P*<0.0001.

As shown in **Figure 7**, on cell scratching assay, cell migration was apparently inhibited on ^131^I-FAPI with cell migration rate (28.38 ± 7.35) %. There were significant differences in the mobility rate between the ^131^I-FAPI group and the ^131^I group ((62.75 ± 3.01) %) or FAPI group ((13.09 ± 3.10) %) the PBS group ((67.02 ± 1.36) %). The migration ability of PANC-1 cells was (29.89 ± 4.52) %, which was significantly lower than that of U87 cells (67.02 ± 1.36) % (*P*<0.001). For PANC-1 cells, migration rate between ^131^I-FAPI group and PBS group has statistical difference (*P*<0.05).

## 4 Discussion

Tumor microenvironment (TME) plays an important role in tumor development, especially in tumorigenesis and metastasis. Fibroblasts are thought to greatly influence the TME (14). The appearance of cancer-associated fibroblast (CAFs) may be responsible to the crosstalk between tumorigenic cells and fibroblasts (15). CAFs are highly heterogenous and can enhance cell migration, alter the metabolism of epithelial tumor cells (16), promote angiogenic cytokine signal (17, 18), regulate the plasticity of cancer cells (19).

FAP derives from CAFs of tumor stroma. It highly expresses in more than 90% epithelial tumors with low or no expression in begin tumors and normal tissues. Molecular probes targeting FAP could achieve high image contrast and low uptake of non-target tissues. In a word, radiolabeled molecule targeting FAP can be specifically used nuclide imaging and treatment of tumors.

FAPI and its analogues, small molecule inhibitors targeting FAP, have been developed and utilized. Molecular probe targeting FAPI are currently used in clinical practice to diagnose and manage a variety of malignances and the metastases. FAPI labeled with ^68^Ga and ^18^F had good diagnostic performances in cancers or sarcoma of head, digestive system, breast and so on. Compared with ^18^F-FDG, these probes are more sensitive in identifying primary tumors and lymph node, bone and visceral metastases (20–22). ^99m^Tc-FAPI is more accessible due to the low cost of ^99m^Tc, and this type of probes is a promising option when PET imaging is not available (7). A series of evaluations on FAPI variants labeled with therapeutic radionuclides, such as ^131^I, ^90^Y, ^177^Lu and ^225^Ac have shown great clinical value on targeted therapy of tumors. The evaluations showed that ^131^I-FAPI-04 and ^177^Lu-EB-FAPI-B1can significantly inhibit U87 glioma (13, 23), ^177^Lu-FAPI-46 and ^225^Ac-FAPI-46/04 had tumor growth inhibition in tumor-bearing models of pancreatic cancer (11, 24). The therapeutic effects were produced through ionizing radiation of radiolabeled FAPIs and resulted in indirect damage to cancer cells adjacent to CAF through crossfire effect (25). Therefore, further research is of great significance to improve the therapeutic efficacy of FAP-targeted radionuclides, including optimizing the chemical structure of FAPI vectors, increasing the dose of therapeutic radionuclides, and combining with other types of therapies (e.g., immunotherapy, external beam radiotherapy, and molecularly targeted therapy).

In this study, tyrosine-modified FAPI was labeled with ^131^I. ^131^I-FAPI was identified as having high stability and hydrophily. In addition, it can be accumulated by tumor cells and inhibit the proliferation and migration of tumor cells through *in-vitro* cell uptake experiments, cell proliferation and cytotoxicity essays and scratching assay. In a word, ^131^I-FAPI was a promising theranostics molecular probe.

The structure of FAPI-04 was selectively modified to promote reaction with ^131^I. FAPI-04 has an N-(4-quinolinyl)-Gly-(2-cyanopyrrolidine) structure that exhibits high nanomolar affinity for FAP and high selectivity for dipeptidyl peptidase and prolyl oligopeptidase (26, 27). The characteristics lead to strong accumulation at tumor sites, low uptake in normal tissues and rapid clearance from circulation of FAPI-04 and its derivatives (28, 29), which are the requisite of high-contrast images.

The chloramine T method was used to label FAPI with ^131^I. The chloramine T method is a commonly direct labeling method. The will-be-used reagents are inexpensive and easy to be obtained, and the steps are highly repeatable. Chloramine T acts as an oxidizing agent and turns the iodine anion into an active iodine molecule. The radiolabeling with ^131^I relies on oxidation reaction. The tyrosine of peptide is essential in this reaction (the widely used method is Chloramine T method). In the radiolabeling reaction, the iodine anion is turned to an active iodine molecule through an oxidizing agent, Chloramine T. Then the hydrogen at the hydroxyl site of tyrosine phenylphene will be replaced by the iodine molecule, resulting in the formation of carbon-iodine chemical bond. In this study, the following conditions are necessary for stable labeling rate at more than 80%. ① The iodine source is fresh, the activity is 5-10 mCi/50 μl. ② Chloramine T is sealed and stored in the dark, and dissolved when it will be used. ③ The volume of iodination reaction is controlled at 250-350μl. We found that the labeling rate of ^131^I-FAPI also increased with the increase of FAPI amount. However, the radiochemical purity of the probe after 24h of the labeling was much lower than that of 0 h when FAPI amount greater than 70μg. Therefore, we selected a FAPI amount with a relatively stable labeling rate for subsequent probe synthesis, i.e., 70μg. Under optimal labeling conditions, the radiochemical purity varied in different environments. The labeling rate in normal saline was significantly lower than that in PBS, so in subsequent experiments, we used PBS for the dilution of ^131^I-FAPI. In the stability test, we found that the probe degraded, because the carbon-iodine chemical bond is not stable and the bond is fragile in different environments.

The lipid-water partition coefficient of the probe was -0.869 ± 0.025, which means that the probe has hydrophilic properties. The result was similar to those FAPIs labeled with ^177^Lu (30), ^68^Ga (31) and ^99m^Tc (32) and slightly different from the lipophilic properties of FAPI labeled with ^131^I (13). In this study, the labeling rate of ^131^I-FAPI was (84.9% ± 1.02) %. When the lipid-water partition coefficient was determined, the freely unlabeled iodine molecules were dissolved in PBS, which increased the radioactivity count in the aqueous phase and correspondingly decreased the value of logP. The hydrophilicity of ^131^I-FAPI referred to that the probe was excreted from urinary system and less bound to plasma proteins, which was conducive to further tumor target therapy (13).

In order to evaluate the retention capacity of ^131^I-FAPI in tumors, we measured the cell uptake of this molecular probe within tumor cells after 24h. U87MG has been used to evaluate the biocharacteristics of FAPIs labeled with ^99m^Tc (32), ^18^F (33) and ^177^Lu (30). And U87MG cell lines was proved to be FAP-positive (33). In our study, U87 cells had the highest uptake to ^131^I-FAPI, which was much greater than that in the blocking group and the ^131^I group (*P*<0.05). In blocking group, FAPI can bind to the FAP receptors that are originally bound with ^131^I-FAPI, which resulted in that the cell uptake rate of blocking group is much lower than that of ^131^I-FAPI group. The statistically significant differences of cell uptake rate between ^131^I-FAPI group and any other groups indicated that ^131^I-FAPI could be specifically accumulated by U87 tumor cells. Compared to ^131^I-FAPl groups, FAPl group had a higher impact on the migration of u87 cells, we sorted out several factors: Firstly, the uptake of the radiolabeled probe might be different from that of the non-radiolabeled peptide, possibly affecting signaling pathways involved in cell migration. Secondly, it is possible that the FAPl remains higher affinity to its target receptors on U87 cells, thus having a more effect on migration. Thirdly, we also consider the possibility of differences in cell culture conditions and timing, which may contribute to the observed differences. PANC-1 cells are a type of epithelial tumor of human pancreatic cancer and our group previously verified the negative expression of FAP in this type of cell lines (8). ^177^Lu-FAPI-46 and ^225^Ac-FAPI-46 could be specifically accumulated by FAP-transfected PANC-1 cells (24). In this study, PANC-1 cells have a certain binding ability to free Na^131^I, and FAPI failed to block the uptake of ^131^I-FAPI by receptors, which indirectly indicates that ^131^I-FAPI has low specificity for PANC-1 cells.

Therapeutic effect of ^131^I-FAPI on tumors was also speculated in this study. The inhibitory effect of the probe on cell proliferation and migration was verified by CCK8 assay and scratching assay. CCK8 assay showed that the proliferation of U87 cells were significantly inhibited in ^131^I-FAPI group and the inhibitory effect gradually increased with time. However, we found that the OD value was abnormally increased after 72 h of drug intervention (not listed in the results). We analyzed that cell damage increased with the intervention of reagents over time, then the cell membrane was broken and the. Or the space and nutrition in the wells were no longer enough to support the cells to proliferate, which resulted in the rupture of cells. The above-mentioned damage brought about the drainage of intracellular dehydrogenase that reacted with CCK8 reagent to produce brownish-yellow formazan. The OD values of PANC-1 cells were irregular because that ^131^I-FAPI was accumulated with low specificity. Cell scratching assay showed that the change of scratching area after 24h incubation was apparently shown in U87 cells. We analyzed that the shape of U87 cells is epithelial cell-like, spindle-shaped with long synapsis, while the shape of PANC-1 cells is oval with limited migration range. The inhibition of ^131^I-FAPI and FAPI on the migration of U87 cells was also obviously shown, which exhibited the specificity of ^131^I-FAPI.

In summary, this study synthesized a novel molecular probe targeting FAP. ^131^I-FAPI has good stability *in vitro* and has hydrophilic properties. *In vitro* cell experiments showed that the probe had good targeting properties and inhibitions on tumor cell proliferation and migration.

There are still some shortcomings and limitations in this study, which were mainly reflected in: ① The properties of the probe was only identified at the cellular level. ② Only two tumor cells were used to identify the target specificity of ^131^I-FAPI. In the future, we will further use other tumor cells and perfect animal experiments in order to develop a therapeutic probe that is more suitable for primary hospitals.

## Data Availability

The original contributions presented in the study are included in the article/Supplementary Material. Further inquiries can be directed to the corresponding author.
