# Corrigendum: Radiosynthesis and *in-vitro* identification of a molecular probe ^131^I-FAPI targeting cancer-associated fibroblasts

**DOI:** 10.3389/fonc.2024.1501235

**Published:** 2024-10-17

**Authors:** Yaxin Tian, Yanghongyan Jiang, Ping Ma, Xiaowei Ma, Liang Du, Fengkui Wang, Xiaodong Yu, Qian Zhao

**Affiliations:** ^1^ Department of Nuclear Medicine, General Hospital of Ningxia Medical University, Yinchuan, Ningxia, China; ^2^ School of Clinical Medicine, Ningxia Medical University, Yinchuan, Ningxia, China; ^3^ Department of Nuclear Medicine, The Second Affiliated Hospital of Guangzhou Medical University, Guangzhou, Guangdong, China

**Keywords:** cancer-associated fibroblasts, FAPI, molecular probe, ^131^I, identification

In the published article, there was an error in affiliation 3. Instead of “Department of Nuclear Medicine, The First Affiliated Hospital of Guangzhou Medical University, Guangzhou, Guangdong Province, China”, it should be “Department of Nuclear Medicine, The Second Affiliated Hospital of Guangzhou Medical University, Guangzhou, Guangdong, China”.

The authors apologize for this error and state that this does not change the scientific conclusions of the article in any way. The original article has been updated.

